# Global status of tetracycline resistance among clinical isolates of *Vibrio cholerae*: a systematic review and meta-analysis

**DOI:** 10.1186/s13756-021-00985-w

**Published:** 2021-08-06

**Authors:** Mohammad Hossein Ahmadi

**Affiliations:** Department of Microbiology, Faculty of Medicine, Shahed University, Tehran, Iran

**Keywords:** *Vibrio cholerae*, Tetracycline, Doxycycline, Resistance

## Abstract

**Background:**

There has been an increasing resistance rate to tetracyclines, the first line treatment for cholera disease caused by *V. cholera* strains, worldwide. The aim of the present study was to determine the global status of resistance to this class of antibiotic among *V. cholera* isolates.

**Methods:**

For the study, electronic databases were searched using the appropriate keywords including: ‘*Vibrio*’, ‘*cholera*’, ‘*Vibrio cholerae*’, ‘*V. cholerae*’, ‘resistance’, ‘antibiotic resistance’, ‘antibiotic susceptibility’, ‘antimicrobial resistance’, ‘antimicrobial susceptibility’, ‘tetracycline’, and ‘doxycycline’. Finally, after some exclusion, 52 studies from different countries were selected and included in the study and meta-analysis was performed on the collected data.

**Results:**

The average resistance rate for serogroup O1 to tetracycline and doxycycline was 50% and 28%, respectively (95% CI). A high level of heterogeneity (*I*^2^ > 50%, *p*-value < 0.05) was observed in the studies representing resistance to tetracycline and doxycycline in O1 and non-O1, non-O139 serogroups. The Begg’s tests did not indicate the publication bias (*p*-value > 0.05). However, the Egger’s tests showed some evidence of publication bias in the studies conducted on serogroup O1.

**Conclusions:**

The results of the present study show that the overall resistance to tetracyclines is relatively high and prevalent among *V. cholerae* isolates, throughout the world. This highlights the necessity of performing standard antimicrobial susceptibility testing prior to treatment choice along with monitoring and management of antibiotic resistance patterns of *V. cholerae* strains in order to reduce the emergence and propagation of antibiotic resistant strains as well as the failure of treatment.

## Introduction

Cholera is an ancient infectious disease mainly affecting developing countries. The disease is capable to spread across many countries leading to vast pandemics and becoming a major public health concern throughout the world [1].

The causative agent of this life threatening diarrheal disease is *Vibrio cholerae* secreting the cholera toxin. Two major cholera toxin-producing serogroups of this bacterial pathogen, O1 and O139, have potential to spread and cause epidemic as well as pandemic disease [2]. The serogroup O1 has two biotypes, classical and El Tor, and each biotype has three serotypes including Ogawa, Inaba, and Hikojima [1, 3].

The main stay of management of cholera (acute gastroenteritis) is urgent fluid replacement; however, the use of an appropriate antibiotic is necessary to eliminate the bacteria, lessen the duration of illness, and control the disease [4].

Tetracyclines (tetracycline and doxycycline) have long been the antibiotics of choice for treating severe cholera effectively worldwide, except for young children and pregnant women [5]. However, tetracycline resistant strains of *V. cholerae* are being increasingly reported worldwide. These resistant strains have been responsible for major epidemics in some countries and geographical areas such as Latin America, Tanzania, Bangladesh, and Zaire [6].

To date, numerous studies have reported the different antibiotic resistance patterns for *V. cholerae* isolates throughout the world. Nevertheless, the overall status of the resistance to teracyclines among the strains is not intensively studied.

The present study was conducted to determine the global status of resistance to the antibiotics of tetracycline family, including tetracycline and doxycycline, among different *V. cholerae* isolates using a systematic review and meta-analysis according to the Preferred Reporting Items for Systematic Reviews and Meta-Analyses (PRISMA) statement [7].

## Methods

### Search strategies

The electronic databases, including OVID databases, PubMed, Web of Science, Scopus, MEDLINE, EMBASE, Cochrane Library, as well as Google Scholar, were searched for papers reporting the resistance rate for different *Vibrio cholerae* isolates to the antibiotics of tetracyclines family from December 1980 to April 2020. The search was restricted to original research articles throughout the world, published in English using the following keywords with the help of Boolean operators (AND, OR): ‘*Vibrio*’, ‘*cholera*’, ‘*Vibrio cholerae*’, ‘*V. cholerae*’, ‘resistance’, ‘antibiotic resistance’, ‘antibiotic susceptibility’, ‘antimicrobial resistance’, ‘antimicrobial susceptibility’, ‘tetracycline’, and ‘doxycycline’. References from reviewed articles were also searched for more information.

### Inclusion and exclusion criteria

Included studies were all original research articles as well as some letter to editors presenting the resistance rates for *Vibrio cholerae* isolates to the tetracyclines including tetracycline and doxycycline.

Excluded articles were those that: (1) had no sufficient data to be analyzed; (2) reported antibiotic resistance of *Vibrio* species other than *V. cholerae*; (3) studied resistance to antibiotics other than tetracyclines; and (4) tested environmental isolates of the bacterium instead of clinical ones; for example, the strains isolated from wastewater, water supplies, river, aquaculture water, fishery products, as well as seafood.

Review articles, congress abstracts, studies reported in languages other than English, meta-analyses or systematic reviews, duplicate publications of the same study and articles available only in abstract form were also excluded.

### Data extraction

The data extracted from each study included first author's name, year of publication, geographical area of study (country), clinical sample (specimen type), serogroup, biotype, and serotype of the isolates, number of investigated isolates (sample size), method of susceptibility testing, and number of isolates resistant to each antibiotic.

### Statistical analysis

The data were analyzed using Comprehensive Meta-Analysis Software Version 2.0 (Biostat, Englewood, NJ, USA). The resistance rate was reported by 95% confidence intervals (CIs).

Cochrane Q-statistic test and *I*^2^ test were performed to estimate heterogeneity between studies, and in all calculations of which *I*^2^ was above 50%, the random effect model was chosen to estimate the average rate because of its conservative summary estimate; otherwise, the fixed effect model was applied. To assess possible publication bias, a funnel plot, Begg’s rank correlation and Egger’s weighted regression methods were used. Two-tailed *p* < 0.05 was considered indicative of a significant publication bias. The relative weight for each study was also calculated.

## Results

**Fig. 1:**
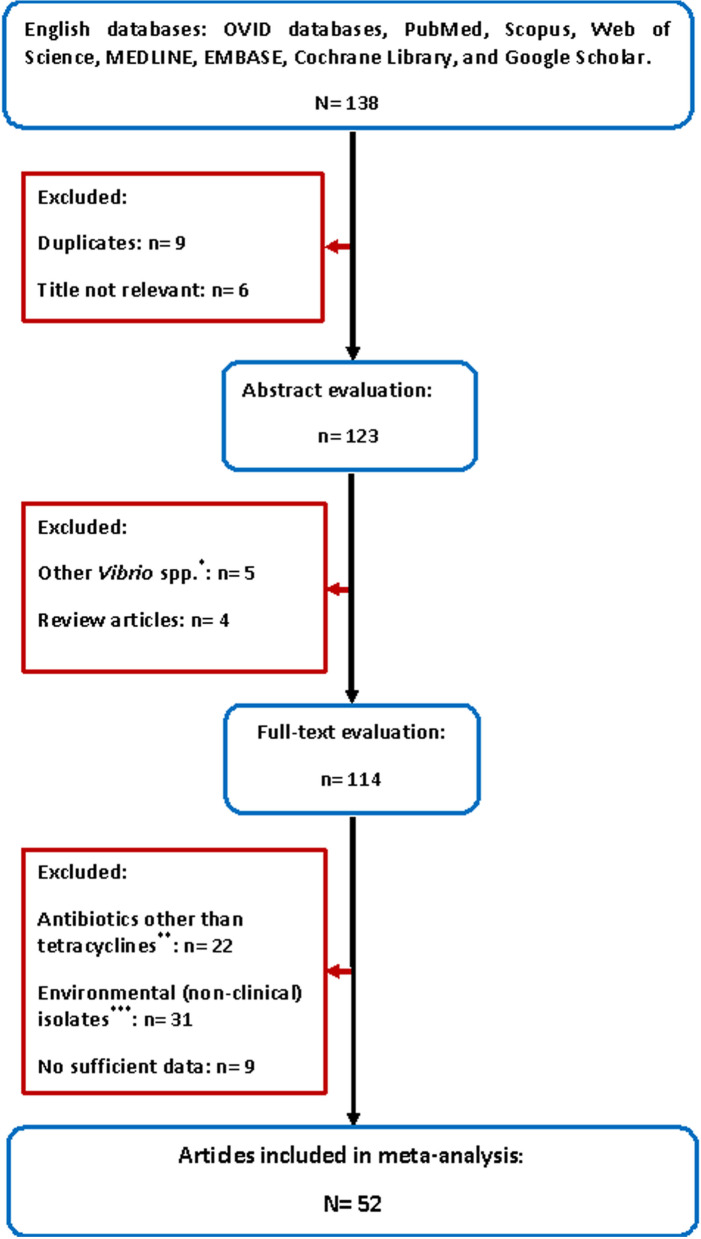
Flow chart of the literature search, systematic review and study selection. *Articles representing study of *Vibrio* species other than *V. cholerae*; **Articles reported resistance to antibiotics other than tetracyclines; ***Studies using environmental instead of clinical isolates of *V. cholerae*

A total of 138 articles were collected for assessment. Through the first screening, 15 articles were excluded on the basis of the title evaluation, as nine of them were duplicate publications of the same study, and six have titles irrelevant to the present study. By the second assessment, nine papers were discarded because they had represented the study of *Vibrio* species other than *V. cholerae*, or were review articles. Finally, after full-text evaluation, 62 studies were ruled out because they had reported resistance to antibiotics other than tetracyclines, used environmental isolates of the bacterium instead of clinical ones, and/or had no sufficient data. Therefore, 52 articles published between 1980–2020 were selected and included in the final analysis (Fig. 1 and Table 1).

**Table 1:** Studies included in meta-analysis after final evaluation

References	Pub. year	Country	Source	Serogroup/biotype	Serotype	No. of isolates	No. of resistant isolate (%)		Method of susceptibility testing
							TET	DOX	
Olipher et al. [8]	2020	Kenya	Stool	Non-O1	ND	98	64 (65.3)	NM	Kirby–Bauer disk diffusion
Kale et al. [9]	2020	India	Stool	O1/El Tor	Ogawa	109	0 (0)	0 (0)	Kirby–Bauer disk diffusion
Abana et al. [10]	2019	Ghana	ND	O1/El Tor	Ogawa	40	14 (35)	6 (15)	Kirby–Bauer disk diffusion
Zereen et al. [11]	2019	Bangladesh	Stool	ND/ND	ND	3	2 (66.7)	NM	Kirby–Bauer disk diffusion
Sreedhara and Mohan [12]	2019	India	Stool	O1/El Tor	Ogawa	74 TET, 41 DOX	19 (25.7)	10 (24.4)	Kirby–Bauer disk diffusion
Dua et al. [13]	2018	India	Stool	Non-O1, non-O139	ND	71	29 (40.9)	63 (88.7)	Kirby–Bauer disk diffusion
Uddin et al. [14]	2018	Bangladesh	Stool	O1/ND	43 Ogawa & 15 Inaba	58	17 (29.3)	10 (17.2)	Kirby–Bauer disc diffusion
Fernández-Abreu et al. [15]	2017	Cuba	Stool	Non-O1, non-O139	ND	125	5 (4)	1 (0.8)	Kirby–Bauer disk diffusion
Shah et al. [16]	2017	Pakistan	Stool & vomitus	ND/ND	ND	131	13 (9.9)	NM	Kirby–Bauer disk diffusion
Dengo-Baloi et al. [17]	2017	Mozambique	Rectal swabs	O1/El Tor	Ogawa	159	79 (50)	89 (56)	Kirby–Bauer disk diffusion
Patil et al. [18]	2017	India	Stool	O1/El Tor	Ogawa	106	10 (9.4)	NM	Kirby–Bauer disk diffusion
Jain et al. [19]	2016	India	Rectal swabs	O1/El Tor	Ogawa	27	27 (100)	NM	Kirby–Bauer disk diffusion & broth dilution
Hajia et al. [20]	2016	Iran	ND	O1/ND	Ogawa & Inaba	192	115 (59.9)	NM NM	Liofilchem Test Strip
Torane et al. [1]	2016	India	Stool	O1/El Tor	407 Ogawa & 32 Inaba	439	55 (12.5)	NM	Kirby–Bauer disk diffusion
Gupta et al. [21]	2016	Nepal	Stool	O1/El Tor	Ogawa	31	0 (0)	0 (0)	Agar dilution
Masoumi-Asl et al. [22]	2016	Iran	Stool	O1/ND	Inaba	60	60 (100)	NM	Liofilchem Test Strip
Irfan et al. [23]	2016	Pakistan	Stool	Non-O1, non-O139	ND	233	5 (2.1)	NM	Kirby–Bauer disk diffusion
Afzali et al. [24]	2016	Iran	Stool	Non-O1, non-O139	ND	96	3 (3.1)	3 (3.1)	Kirby–Bauer disk diffusion
Kar et al. [6]	2015	India	Rectal swabs	O1/El Tor	Ogawa	35	35 (100)	NM	Kirby–Bauer disk diffusion
Ukaji et al. [25]	2015	Nigeria	Stool	O1/ND	ND	63	53 (84.1)	NM	Kirby–Bauer disk diffusion
Tabatabaei and Khorashad [26]	2015	Iran	Stool	O1/ND	Inaba	48	29 (60.4)	NM	Kirby–Bauer disk diffusion
Barati et al. [27]	2015	Iran	Rectal swabs	O1/El Tor	Ogawa	239	36 (15.1)	10 (4.2)	Kirby–Bauer disk diffusion
Mishra et al. [28]	2015	India	Stool	O1/El Tor	Ogawa	44	0 (0)	NM	Kirby–Bauer disk diffusion
Kuma et al. [29]	2014	Ghana	Stool & vomitus	O1/ND	ND	275	43 (15.6)	40 (14.5)	Kirby–Bauer disk diffusion
Mercy et al. [30]	2014	Kenya	ND	O1/El Tor	Inaba (most commen) & Ogawa	44	0 (0)	0 (0)	Kirby–Bauer disk diffusion
Mahmud et al. [31]	2014	Sierra Leone	Rectal swabs	O1/El Tor	Ogawa	15	0 (0)	0 (0)	Kirby–Bauer disk diffusion & E-test
Murhekar et al. [32]	2013	Papua New Guinea	Stool & rectal swabs	O1/El Tor	Ogawa	299	29 (9.7)	NM	Kirby–Bauer disk diffusion
Tran et al. [33]	2012	Vietnam	ND	O1/El Tor	Ogawa	100	29 (29)	0 (0)	E-test strips
Sang et al. [34]	2012	Kenya	Stool	O1/ND	ND	4	0 (0)	NM	Kirby–Bauer disk diffusion
Mandal et al. [35]	2012	India	Stool	O1/El Tor	150 Ogawa & 4 Inaba	154	26 (16.9)	NM	Agar dilution & E-test
Shujatullah et al. [36]	2012	India	Stool	O1/ND	Ogawa (most commen) & Inaba	66	9 (13.6)	8 (12.1)	Kirby–Bauer disk diffusion
Borkakoty et al. [37]	2012	India	Rectal swabs	O1/El Tor	24 Ogawa & 16 Inaba	40	16 (40)	NM	Kirby–Bauer disk diffusion
Das et al. [38]	2011	India	Stool & rectal swabs	O1/ND	Ogawa (most common), Inaba, Hikojima	238	41 (17.2)	NM	Kirby–Bauer disk diffusion & broth dilution
Karki et al. [39]	2011	Nepal	Stool	O1/El Tor	Ogawa	57	0 (0)	NM	Kirby–Bauer disk diffusion
Rahbar et al. [40]	2010	Iran	Stool & rectal swabs	O1/El Tor	199 Inaba & 21 Ogawa	220	0 (0)	0 (0)	Kirby–Bauer disk diffusion
Abera et al. [41]	2010	Ethiopia	Stool	O1/ND	Inaba	81	5 (6.2)	0 (0)	Kirby–Bauer disk diffusion
Supawat et al. [42]	2009	Thailand	Stool & rectal swabs, blood	O1/ND O139 Non-O1, non-O139	1032 Inaba & 43 Ogawa ND ND	1075 41 22	16 (1.5) NM 3 (13.6)	NM NM NM	Kirby–Bauer disk diffusion
Keramat et al. [43]	2008	Iran	Stool	O1/El Tor	Inaba	60	14 (23.3)	20 (33.3)	Kirby–Bauer disk diffusion
Roychowdhury et al. [44]	2008	India	Stool & rectal swabs	O1/ND	Inaba & Ogawa	51	9 (17.6)	NM NM	Kirby–Bauer disk diffusion
Mandomando et al. [45]	2007	Mozambique	Rectal swabs	O1/ND	Ogawa	75	73 (97.3)	NM	Kirby–Bauer disk diffusion
Faruque et al. [5]	2007	Bangladesh	ND	O1/ND	762 Ogawa & 535 Inaba	1297	711 (54.8)	NM	Kirby–Bauer disk diffusion
Rafi et al. [46]	2004	Pakistan	Stool	O1/ 66 El Tor & 57 Classical	ND	123	37 (30.1)	NM	Kirby–Bauer disk diffusion
Tjaniadi et al. [47]	2003	Indonesia	Stool & rectal swabs	O1/ND Non-O1, non-O139	ND ND	1044 68	12 (1.2) 6 (8.8)	NM NM	Kirby–Bauer disk diffusion
Dromigny et al. [48]	2002	Madagascar	Stool	O1/El Tor	ND	351	55 (15.7)	NM	Kirby–Bauer disk diffusion
Sabeena et al. [49]	2001	India	Stool	O1/El Tor	Ogawa	25	2 (8.0)	NM	Kirby–Bauer disk diffusion
Iwanaga et al. [50]	2000	Laos	ND	O1/El Tor	Ogawa	99	95 (95.9)	NM	Agar dilution
Urassa et al. [51]	2000	Tanzania	Stool	O1/ND	ND	181	42 (23.2)	NM	Kirby–Bauer disk diffusion
Garg et al. [52]	2000	India	ND	O1/ND O139 Non-O1, non-O139	Ogawa ND ND	326 314 200	8 (2.5) 8 (2.5) 55 (27.5)	NM NM NM	Kirby–Bauer disk diffusion
Ranjit et al. [53]	2000	Malaysia	ND	ND/ND	ND	24	8 (33.3)	NM	Kirby–Bauer disk diffusion
Dhar et al. [54]	1996	Bangladesh	Stool	O1/El Tor O139	ND ND	110 132	46 (42) 0 (0)	1 (0.9) 0 (0)	Kirby–Bauer disk diffusion
Ng and Taha [55]	1994	Malaysia	Rectal swabs	O1/El Tor	Ogawa	3	3 (100)	NM	Kirby–Bauer disk diffusion
Glass et al. [56]	1980	Bangladesh	Stool	O1/ND	Inaba & Ogawa	256	54 (21.1)	NM	Kirby–Bauer disk diffusion & broth dilution

*NM* not measured; *ND* not determined; *TET* tetracycline; *DOX* doxycycline

The included studies were carried out in 20 different countries, majority of which (38 studies) located in Asia, 12 in Europe, one in Caribbean, and one in Oceania (Table 1).

Of 52 articles included, 40 had studied only O1 serogroup all of which reported El Tor biotype, five had detected only non-O1, non-O139 serogroup, four had investigated O1, O139, and/or non-O1, non-O139 serogroups simultaneously, and three did not determined the serogroups of isolated *V. cholerae*. From the included studies, only one had detected and tested classical biotype beside the El Tor one.

The most commonly collected samples for assessment in the included studies were stool, and rectal swabs, but other samples included vomitus and blood (for isolation of non O1, non O139 *V. cholera*e).

The included studies tested antimicrobial susceptibility to tetracycline and doxycycline for the serogroups O1 (44 and 16 studies, respectively), O139 (two and one studies, respectively), and non-O1, non-O139 (eight and three studies, respectively) of *V. cholerae*. From the studies conducted on serogroup O1, 19 detected only Ogawa, three only Inaba, and 12 detected both serotypes, simultaneously. Only one study detected Hikojima serotype along with other two serotypes, concurrently. The remaining studies conducted on serogroup O1 did not determine the serotypes of their isolates.

The studies mainly used Kirby-Bauer disk diffusion method for susceptibility testing, but other techniques were broth and agar dilution, E-test, and Liofilchem Test Strip.

The number of *V. cholerae* isolates investigated (sample sizes) in the studies varied from 3–1297. The range of antibiotic resistance as well as the pooled resistance rate for *V. cholerae* isolates (serogroups O1, O139, and non-O1, non-O139) to tetracycline and doxycycline are shown in Table 2.

**Table 2:** Meta-analysis results for resistance rate of each *V. cholera* serogroup in included studies

Serogroup	Number of studies	Antibiotic	Resistance rate (%) (95% CI)			Heterogeneity test		Begg’s test** *p*-value (two-tailed)		Egger’s test*** *p*-value (two-tailed)
			Min	Max	Pooled* (range)	*I*^2^ (%)	*p*-value	a	b	
O1	44 16	TET DOX	0 0	100 56	0.2 (0.1–0.3) 0.07 (0.03–0.1)	96.9 92.5	< 0.001 < 0.001	0.2 0.7	0.2 0.8	0.03 0.005
O139	2 1	TET DOX	0 NA	2.5 NA	0.02 (0.01–0.04) 0.004 (0.0–0.06)	43 0.0	0.2 1.0	NA NA	NA NA	NA NA
Non-O1, non-O139	8 3	TET DOX	2.1 0.8	65.3 88.7	0.1 (0.05–0.3) 0.1 (0.001–0.9)	95.5 97.7	< 0.001 < 0.001	0.2 0.6	0.3 1.0	0.06 0.3

*TET* tetracycline; *DOX* doxycycline; a: Kendall’s tau without continuity correction; b: Kendall’s tau with continuity correction; *Pooled resistance rate; **Begg and Mazumdar rank correlation; ***Egger’s regression intercept; NA: not applicable

**Fig. 2:**
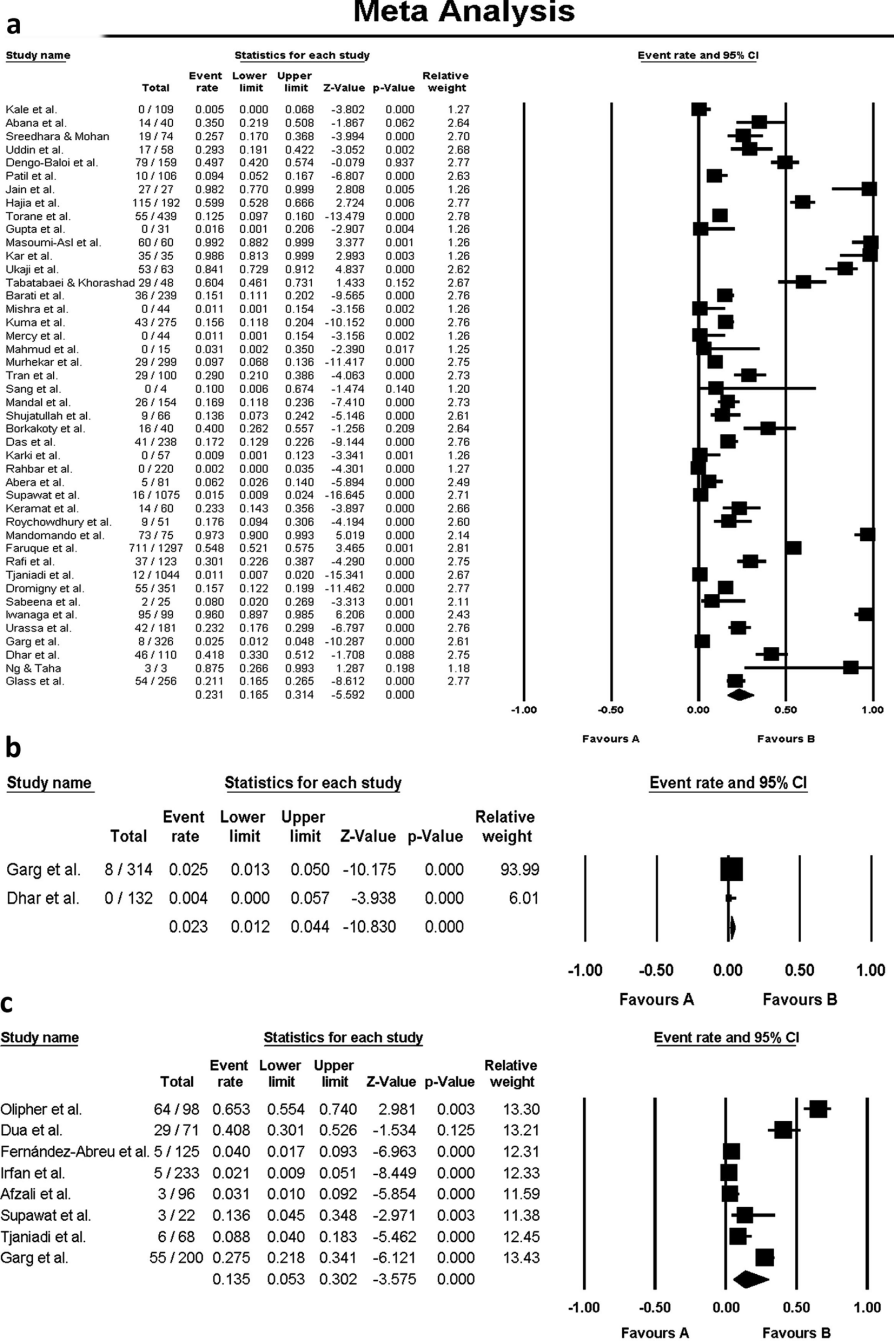
Forest plots of the meta-analysis for resistance rate of *V. cholerae* serogroups O1 (**a**), O139 (**b**), and Non-O1, non-O139 (**c**) to tetracycline

**Fig. 3:**
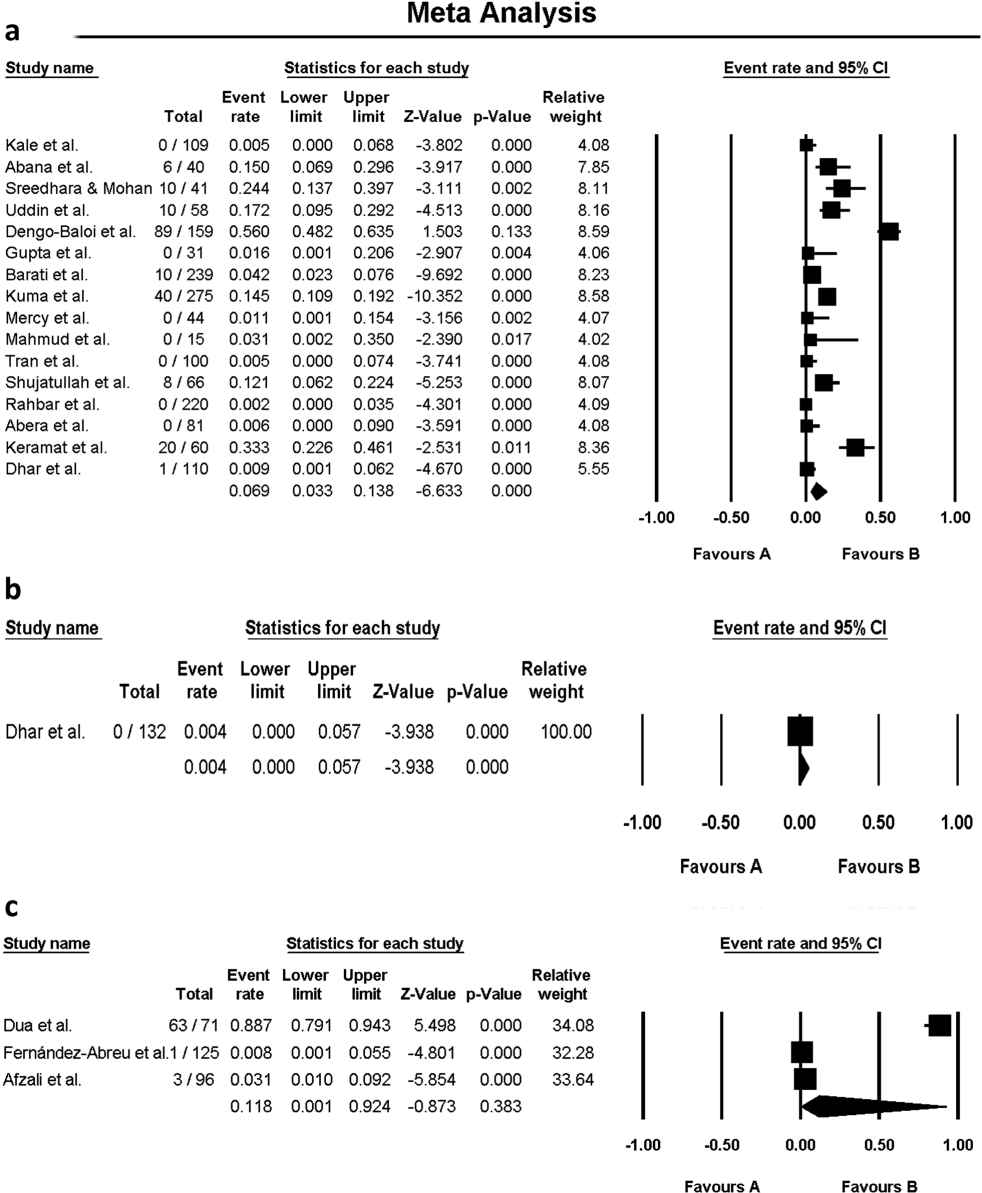
Forest plots of the meta-analysis for resistance rate of *V. cholerae* serogroups O1 (**a**), O139 (**b**), and Non-O1, non-O139 (**c**) to doxycycline

The average resistance rate for serogroup O1 to tetracycline and doxycycline was 50% and 28%, respectively (95% CI). Figures 2a–c and 3a–c show the forest plots of the meta-analysis for resistance rate of different serogroups of *V. cholerae* to the antibiotics. A high level of heterogeneity (*I*^2^ > 50%, *p*-value < 0.05) was observed in the studies representing resistance to tetracycline and doxycycline in O1 and non-O1, non-O139 serogroups; however, the number of included studies conducted on the antimicrobial resistance of O139, as well as non-O1, non-O139 serogroups to tetracycline and doxycycline was fewer than 10 and insufficient for an accurate analysis.

**Fig. 4:**
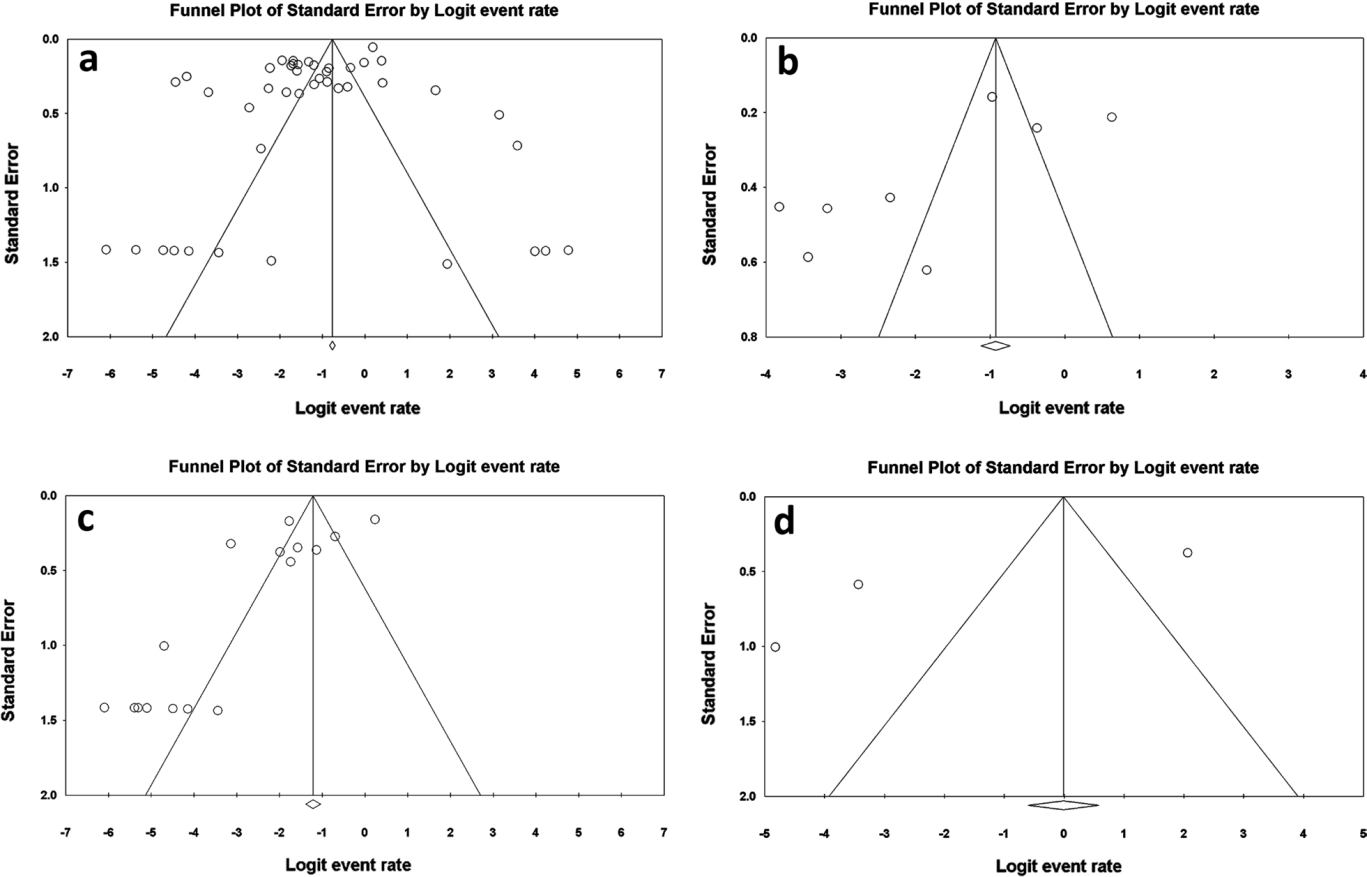
Funnel plots of the meta-analysis for resistance rate of *V. cholerae* serogroups O1 and Non-O1, non-O139 to tetracycline (**a**, **b**) and doxycycline (**c**, **d**), respectively. (In case of serogroup O139, the number of included studies was fewer than three and insufficient for application of funnel plot)

The Begg’s tests did not indicate the publication bias (*p*-value > 0.05). However, the Egger’s tests showed some evidence of publication bias in the studies conducted on serogroup O1 (Table 2). The corresponding funnel plots of the all the analyses (except serogroup O139 in which the number of included studies was fewer than three and insufficient for application of funnel plot), are shown in Fig. 4a–d.

## Discussion

The historical disease, cholera, has been endemic in south Asia, especially the Ganges delta region in Bangladesh and India, from which the disease spread outside the Indian subcontinent along trade routes causing the pandemics with high mortality rates (millions of deaths) throughout the world [2]. To date, toxigenic *Vibrio cholerae* (O1 serogroup) has caused seven pandemics, six of which were due to classical biotype and the seventh pandemic caused by El Tor one [37].

Although the antibiotics cannot be used as a sole treatment for the disease; however, combining fluid replacement therapy with antibiotic treatment has advantages as the antibiotics could lessen the duration of illness and reduce shedding of *V. cholerae* in the stool [4].

Tetracyclines are ‘broad-spectrum antibiotics’ that inhibit the bacterial 30S ribosomal subunit and consequent protein synthesis [57]. These antibiotics, particularly tetracycline and doxycycline, have long been the antibiotics of choice for treating severe cholera around the world, except for young children and pregnant women [2, 5]. However, tetracycline-resistant strains of *V. cholerae* have been emerged continuously over the years, due mainly to the extensive clinical and non-clinical uses of this class of antibiotic [6, 52].

By performing this systematic review and meta-analysis, it was found that the resistance rate of *V. cholerae* isolates to tetracyclines was greatly variable in various studies conducted in different geographical areas. The regional differences in resistance rate of *V. cholerae* isolates to tetracyclines may result from various exposure of patients in different populations to the antibiotics. This highlights the necessity of regional and local antibiotic susceptibility testing before antibiotic administration to avoid failure of treatment.

The high level of heterogeneity in the studies as well as the differences in sample sizes might impact on the analyses. To overcome this problem, the relative weight for each study was calculated and considered in the present study. Another problem in the current study was that the number of included studies conducted on the antimicrobial resistance of O139 and non-O1, non-O139 serogroups of *V. cholerae* was fewer than 10 and insufficient for a powerful meta-analysis and an accurate conclusion.

The results of the present meta-analysis showed that the overall resistance rate of *V. cholerae* isolates to tetracyclines (including tetracycline and doxycycline) was relatively high and between these two antibiotics, the average resistance rate to tetracycline was higher in serogroup O1.

Tetracycline resistance in *V. cholerae* isolates has been reported from Bangladesh since 1979 [56]. As with other antibiotics, the genes encoding resistance to tetracyclines commonly locate on mobile genetic elements such as plasmids and transposons by which the genes could be rapidly transferred and exchanged among the clinical as well as environmental strains of *V. cholera*, leading to increased resistance to these antibiotics [58]. Moreover, the antibiotic resistance determinants may be transferred and exchanged between environmental and clinical isolates of *V. cholera* through the horizontal gene transfer mechanisms [59]. These events lead to rapid increase in antibiotic resistance among the isolates.

Among the involved mechanisms of resistance to tetracyclines, the active efflux of antibiotic from bacterial cell as well as the production of ribosomal protection proteins (encoded by *tet* genes) are predominant in clinical settings. The other implicated mechanisms are target site mutation, decreased drug permeability, and enzymatic degradation of the antibiotic [58].

It has been evidenced that classical biotype of *V. cholerae* generally causes more severe illness compared to El Tor counterpart; in turn, the latter biotype is more adaptable and flexible in the environment, has more asymptomatic carriers, and causes higher infection to case ratio [37]. Furthermore, it has been shown that the strains of *Vibrio cholerae* serogroup O1 may change the biotype from Ogawa to Inaba and vice versa. Such biotype interconversion has been linked to variation in antibiotic resistance in some cases [18].

Besides tetracyclines as the first line drugs, the other antibiotic options for treatment of severe cholera include furazolidone, ciprofloxacin, erythromycin, trimethoprim-sulphamethoxazole, and chloramphenicol [5]. However, the emergence of multiple antibiotic resistant strains of *V. cholerae* (displaying resistance against several antibiotics) is a major global issue and a serious problem for public health. The reasons for the appearance and development of such resistant strains may be attributed to the extensive misuse of antibiotics without proper susceptibility testing as well as the lack of an appropriate national surveillance program to monitor the bacterial resistance patterns [6]. For example, the emergence of tetracycline resistant strains causing an epidemic in Tanzania was due to the widespread use of this antibiotic for prophylaxis [50].

On the other hand, in some countries, wastewater and human excreta are routinely used for farming or in the aquaculture systems. This causes the shedding of *Vibrio cholera* to these environments. It is known that antibiotics are also disseminated into the environment in many ways such as excretion from humans or animals (through urine and feces), farming, and/or disposal of antimicrobials. The degradation of some antibiotics including tetracyclines takes a considerably longer time. Therefore, these antibiotics remain in water for a long period of time and gradually accumulate to reach a higher concentration. Consequently, the exposure of *V. cholerae* strains to these antibiotics in environmental settings, may lead to development and increase of resistant strains in aquatic ecosystem through natural selection. Eventually, the aquatic ecosystem as well as aquatic products serve as important reservoirs for antibiotic resistant as well as more virulent *Vibrio cholerae* strains capable to spread and transmit to humans via direct contact or through the food chain [59], thereby causing the epidemic infections characterized by failure in treatment.

The antimicrobial susceptibility testing according to approved CLSI guidelines (M45) is necessary prior to treatment choice [60]. However, it has been revealed that in vitro susceptibility of *V. cholerae* to antibiotics does not necessarily correlate with in vivo activity [3]. Recently, some non-antibiotic techniques have been introduced as possible alternatives to traditional antibiotics in order to control pathogens and minimize the risk of development of antibiotic-resistant strains in the environment. These possible alternatives may include inhibition of bacterial quorum sensing (quorum quenching), application of bacteriophages, and using of probiotics [59]. Moreover, it is notable that some vaccines are currently licensed or under development for prophylaxis against cholera disease in children and adults as reviewed by Shaikh et al. [61].

## Conclusion

In conclusion, the results of the present study show that the overall resistance to tetracyclines, the first line treatment for cholera disease, is relatively high and prevalent among *V. cholerae* isolates, throughout the world. Hence, performing regional antimicrobial susceptibility testing according to approved CLSI guidelines prior to treatment choice along with monitoring and management of antibiotic resistance patterns of *V. cholerae* strains seems to be necessary. In this regard, planning the national or international surveillance programs would be helpful to reduce the emergence and propagation of antibiotic resistant strains as well as the failure of treatment.

## Data Availability

All data generated by this study have been submitted with this manuscript.

## Abbreviations

CI: Confidence interval
PRISMA: Preferred reporting items for systematic reviews and meta-analyses
